# Expert hearings in mini-publics: How does the field of expertise influence deliberation and its outcomes?

**DOI:** 10.1007/s11077-022-09465-3

**Published:** 2022-08-06

**Authors:** Mikko Leino, Katariina Kulha, Maija Setälä, Juha Ylisalo

**Affiliations:** Department of Philosophy, Contemporary History and Political Science, University of Turku, Turku, Finland

**Keywords:** Deliberation, Mini-public, Expert information, Pandemic

## Abstract

One of key goals of deliberative mini-publics is to counteract expert domination in policymaking. Mini-publics can be expected to democratize expertise by providing citizens with good opportunities for weighing expert information. Yet, there are concerns about undue influence of experts even within mini-publics. We test these expectations by analysing data from an online mini-public organized in Finland in March 2021. The topic of deliberation was measures taken to contain the COVID-19 pandemic. We examine whether experts’ field of specialization and the order of expert hearings had an impact on how participants’ views developed. We find that neither the field of expertise nor the order of hearings had systematic effects on participants’ perceptions on containment measures. The results suggest that interactive modes of expert hearings in mini-publics seem not to be prone to domination by experts.

## Introduction

Information and advice provided by scientific or other technical experts can profoundly affect how public policies are formulated, as has been demonstrated by how the COVID-19 pandemic has been handled in many countries (see, for example, Moore & MacKenzie, 2020). Experts represent professional communities specializing in the production of reliable information on a given subject matter, and expertise thus is an important aspect of the division of deliberative or epistemic labour in advanced societies (cf. Richardson, 2002). Obviously, processes of producing expert knowledge—the scientific process, for example—are very different from democratic deliberation required for making legitimate public decisions.

Political decisions require scientific and technical expertise, which has given rise to concerns that political decision making is moving into the hands of unelected policy experts, further away from the citizens and elected representatives (Dahl, 1989, 332–338). The concern over excessive impact of experts in public decision-making was one of the reasons why Dahl (1989, 340–341) first proposed his idea of a mini-populus, which is a randomly selected citizen body complementing representative decision making on complex issues.

One of the key functions of forums for citizen deliberation, or so-called deliberative mini-publics, is to respond to technocratic tendencies in advanced societies by bridging the gap between citizens and experts (cf. Dahl, 1989). Deliberative mini-publics usually feature interaction with experts in order to help participants form more informed and reflected views on the policy issue at hand (Setälä & Smith, 2018). In general, deliberative mini-publics can be expected to offer participants good opportunities for scrutiny and weighing of expert information. However, there are also concerns that participants accept expert views uncritically or that the experts have too much of an influence on how participants’ opinions develop in the course of the deliberative process (e.g. Moore & MacKenzie, 2020).

Research concerning the role of expert information in deliberative mini-publics is still relatively scarce (see also Drury et al., 2021; Roberts et al., 2020; Muradova et al., 2020). While some empirical evidence on expert influence on deliberators’ opinions exists (e.g. Luskin et al., 2002; Setälä et al., 2010), there are no studies systematically examining to what extent expert hearings influence opinion formation in mini-publics and whether these venues provide citizens with good opportunities to evaluate scientific and technical expertise. This article aims to fill part of this gap. We look at whether and how experts heard in deliberative mini-publics affect the ways in which participants’ attitudes change in the course of deliberation. More specifically, we analyze  the impact of expert hearings in a deliberative mini-public on citizens’ perceptions of and views on the containment measures related to the COVID-19 pandemic. Our data are based on a deliberative mini-public that was held online in Finland in March 2021. The topic of deliberation was the measures that had been taken to counter the spread and contain the effects of the pandemic in Finland.

This topic is well-suited for the purpose since experts representing different fields may arguably have quite differing views on the pandemic policies. Moreover, policy responses to the pandemic often exemplify technocratic tendencies in representative systems. Policy decisions have strongly relied on the information provided and proposals made by epidemiologists, medical researchers and other health experts (Moore & MacKenzie, 2020). In addition to medical experts, the views of legal experts, economists and social scientists have influenced public policies and evaluations of their feasibility.

We focus on the following two main research questions. First, how does an expert’s field of expertise affect participants’ attitudes to pandemic policies? Second, are participants’ views affected by the order in which they hear experts of different fields? We found that even though the deliberative process increased participants’ knowledge and changed their opinions, the impacts of the field of expertise and the order of expert hearings appear to have been limited. This finding seems to dispel at least some of the concerns related to excessive expert influence in mini-publics. At the end of the article, we discuss the implications of our results for the design of deliberative mini-publics more generally.

## The role of expert information in deliberative mini-publics

### How can deliberative mini-publics help avoid the risks of undue expert influence?

Deliberative mini-publics are forums gathering a group of randomly selected citizens to deliberate on a given political issue. While they come in a wide variety of forms (see, for example, Setälä & Smith, 2018), there are certain key design features. These are a random selection of participants, moderated small group discussions, and the opportunity for participants to gather evidence and receive information on the issue at hand. The information-gathering may entail information sheets prepared beforehand and expert hearings during the deliberation.

Giving participants an opportunity to acquire evidence enables them to make better informed and well-considered judgements on the policy issues at hand (see Brown, 2014). In this respect, expert information complements the pool of information that participants possess before deliberation. In addition, giving participants access to expert information may help level differences in participants’ deliberative capacities, for example, by making it easier for those with lower levels of education to engage in the deliberative process.

Overall, deliberation can be expected to provide good conditions for information-processing (Warren, 2002, 194–195), and there are reasons to believe that this applies to mini-publics in particular. First, in mini-publics, participants can focus on a single issue for a longer period of time, which provides opportunities for critical reflection. Second, mini-publics allow participants representing different viewpoints to engage in deliberation on various forms of evidence, as well as assess the quality of evidence and arguments given by experts, and witnesses’ credentials and trustworthiness. A deliberative process where people need to justify their views to a diverse group of people is arguably an efficient method of correcting individual biases (Mercier & Landemore, 2012), including in terms of processing expert information.

Nonetheless, processing expert information is challenging even in the context of a mini-public. Expert information is often complex which makes it difficult for lay citizens to understand. More importantly, expertise may create hierarchies that hinder equitable consideration of arguments by their merits. Sometimes lay citizens may make judgements about the experts themselves rather than the contents of their arguments. People often use academic qualifications or similar credentials as shortcuts when assessing the reliability of expert information (Warren & Gastil, 2015). However, judgements regarding expertise may also be based on other factors that should be entirely irrelevant, such as experts’ age, gender, ethnicity or even physical appearance.

From the perspective of deliberative democracy, the main problem with expertise is that it may undermine individuals’ capacity for critical judgement. When there is deference to scientific authority, experts are not just trusted, but their views are regarded as authoritative (Howell et al., 2020). Such deference entails that expert views have a strong impact on how individuals perceive the issue, what kinds of beliefs they have and, consequently, their attitudes and views. This can lead to undue impact of experts, or expert domination, a situation where the participants simply recreate expert opinion. This potential for elite domination in mini-publics has been raised as a potential flaw in their institutional design. For example, it is assumed that “by selection of readings and expert witnesses” organizers can manipulate both the participants’ views and the output of mini-publics (Tucker, 2008, 136).

In addition, citizens may use authoritative expert information highly selectively to confirm their pre-existing views; individuals may use expert information as a ‘weapon’ to defend one’s own arguments. In sum, expertise may foreclose the prospects of democratic deliberation and violate key virtues, such as openness to different viewpoints and critical reflection. At worst, expert hearings that are supposed to be learning processes that enhance participants’ deliberative capacities turn out to be exclusionary processes that suppress the voices of participants (Bogler, 2012).

However, deliberation in mini-publics should counteract such effects because it supposedly encourages participants to exercise their critical capacities. When designing deliberative processes dealing with expert information, the guiding principle should be to support processes of public scrutiny of information (Moore, 2017; Roberts et al., 2020). The key issue is not to just develop two-way communication between experts and lay citizens, but rather procedures that better integrate experts into the deliberative process among citizens.

For example, expert hearings could be made an integral part of small group deliberations (Roberts et al., 2020). Even plenary-type hearings should be preceded and followed by deliberations in pairs or, even more preferably, in small groups. This is likely to foster critical reflection on expert information among participants and help avoid blind deference or selective use of expert information.

### Framing and path-dependency in deliberative processes

Even if such measures are taken in mini-publics, expert information may have a subtler impact by influencing the framing of the issue. According to Chong and Druckman (2007, 104), framing can be defined as “the process by which people develop a particular conceptualization of an issue or reorient their thinking about an issue.” In other words, frames are organizing principles that help people conceptualize issues, evaluate arguments, and connect arguments with values. Framing implies a selection of legitimate concepts and viewpoints as well as emphasizing some issues at the expense of others. Frames involve multiple “forms of evidence, from technical and scientific to personal and moral” (Drury et al., 2021, 31). Such processes have an impact both on the contents of discussion and on individual opinion-formation (Calvert & Warren, 2014). In previous studies, elite groups, such as experts, have been recognized as the key sources of frames in public discussion (Chong & Druckman, 2007).

In the context of deliberative mini-publics, expert hearings provide frames that influence the ways in which an issue is discussed and how participants form their opinions (Barisione, 2012). Frames provided by experts can therefore have a strong impact on how deliberators conceptualize the issue, how they value different arguments and which views they regard as valid. In other words, frames may exclude other possible perspectives on the same matter. As Barisione (2012) notes, this does not necessarily require a formal flaw in the deliberative process.

As pointed out above, from the perspective of deliberative democracy, frames provided by experts should not dominate the discussion and foreclose alternative frames. In addition to enhancing deliberation and critical reflection, the presence of alternative and conflicting frames can help neutralize the dominance of a particular frame in a deliberative mini-public. Indeed, organizers of deliberative mini-publics typically invite several experts to the hearing process to ensure a plurality of expert views. The fact that different experts approach the same phenomena from different perspectives should counteract strong framing effects.

Yet, there is still the prospect that the order of expert hearings may create discursive path-dependencies that can impact the outcome of a deliberation (cf. Goodin, 2008, 115). As Goodin (2000, 88–89) argues, “[f]rom everyday life we know that different conversations with different participants (or with the same participants interjecting at different points) proceed in radically different directions.” Along with framing by experts, the possibility of discursive path-dependency implies that the order of expert hearings may be crucial in determining the direction and outcomes of a discussion.

For example, in their analysis concerning media framing of immigration policy agenda, Dekker and Scholten (2017, 215) note that “the actor that is able to first frame the focusing event is often able to maintain the upper hand.” From this perspective, the experts heard first can define the initial frames of discussion and influence the range of perspectives and interpretations that deliberators deem relevant or irrelevant. This also means that the one who introduces their argument last has to make more effort when presenting their contesting frame.

### Hypotheses

While participants’ opinions are expected to change throughout a deliberative process, the information phase has been considered to be the most influential in opinion change (see, for example, Luskin et al., 2002; Thompson et al., 2021). In our experiment, the information phase consisted of Q&A sessions, where the participants interacted with experts from two major fields: law and the social sciences, on the one hand, and healthcare, on the other. The selection of experts was motivated by the observation that expert opinions on desirable responses to the pandemic had varied considerably. To generalize, legal experts and social scientists had been prone to put more weight on people’s fundamental rights and freedoms and highlight the social problems created by strict containment measures, whereas health experts had tended to be more concerned about the risks related to high infection rates and had therefore been more supportive of strict restrictions (see Moore & MacKenzie, 2020).

Based on previous findings (see, for example, Drury et al., 2021), we assume that participants in a mini-public might not be ready to absorb information and evidence from all fields of expertise equally. Moreover, it is reasonable to expect that the opinions and information provided by experts from different fields invite the participants to take different kinds of issues into consideration, even if the views expressed by the experts were moderate in terms of justifications for or against strict containment measures. As Moore (2017, 150) notes, “where an issue is already politically controversial it is hard for experts to avoid becoming implicated, for even an apparently neutral statement is likely to serve one side over another”. Therefore, regarding our experimental procedure, if the starting point of the discussion is public health, the participants are more likely to think about containment measures in terms of their public health benefits than in terms of the risks they create in other respects, while the adverse is more likely when the starting point is connected to legal and social questions.

In this study, we examine, first, whether the field of expertise had an impact on how participants’ opinions developed and, second, whether the experts heard first had a stronger impact. For these purposes, we manipulated the order in which the participants interacted with experts representing different fields. The three hypotheses below summarize our expectations on how the experimental treatments would affect the development of participants’ opinions on pandemic policies.

#### H1

Hearing of health experts makes participants more supportive of restrictions.

#### H2

Hearing of social/legal experts makes participants more critical of restrictions.

#### H3

The experts heard first have a stronger impact on participants’ views.

In addition to testing these hypotheses, we examined participants’ questions to experts and their evaluations of the experts’ answers in order to provide a fuller account of the impact of the field of expertise and participants’ perceptions of the experts’ performance.

## The experiment: deliberation on COVID-19 policies in Finland

The COVID-19 pandemic has had widespread and profound effects on human lives and entire societies across the world. Not all countries have been hit equally by the pandemic, but it is safe to say that practically no nation has remained unaffected. The pandemic has caused loss of lives and put national healthcare systems under stress, which has made governments adopt drastic measures to contain the spread of the virus. These measures have included, for example, stricter border control or other restrictions on mobility, quarantines for people exposed to the virus, and closings of public venues and private businesses.

Addressing the pandemic is not only about finding the most effective ways of preventing the spread of the virus. All measures taken to this end have to be weighed against the direct and indirect costs that they inflict, their legitimacy in the eyes of the public, and their impact on people’s rights and freedoms. Restrictions have aimed at preventing the loss of lives and relieving the pressure on healthcare systems. At the same time, they have violated citizens’ basic rights, increased state-led surveillance, caused economic losses and increased inter-group inequalities, stirred political and societal unrest and, initially, sparked fears of a global economic downturn (see, for example, Eck & Hatz, 2020; Nicola et al., 2020; Tiirinki et al., 2020). The general public has received the measures with varying sentiments and levels of compliance (Georgieva et al., 2021).

By early 2021, Finland, with its population of 5.5 million and relatively low population density (18.2 per square kilometre on average), had survived the pandemic with fewer deaths and less serious repercussions than many other countries. When a deliberative mini-public was held in March 2021, there were fewer than 80,000 confirmed cases of COVID-19 in Finland, and the pandemic had claimed around 800 lives (Finnish Institute for Health and Welfare, 2021a). The restrictions introduced in 2020–2021 were in many respects loose, by European standards. For example, bars and restaurants remained open most of the time, there had been no legal obligation to wear face masks and, with the exception of a period of less than three weeks in the spring of 2020, people were free to travel within the country.

Still, the restrictions imposed by the government included measures seen in other countries, including, for example, closings of schools, public venues and private businesses, restrictions on entering the country, alongside recommendations related to abstaining from traveling, working from home and reducing social contacts (see Tiirinki et al., 2020). In line with the national coronavirus strategy, the intensity of these measures varied across regions and over time, depending on the local COVID-19 situation. The most severe restrictions were in place in the spring of 2020, while the summer and early autumn of the year can be described as less strict in terms of pandemic containment measures.

Finland exemplifies a high-trust society in terms of general and political trust as well as confidence in public officials (Grönlund & Setälä, 2012), which seems to be associated with high levels of compliance with measures taken to contain the pandemic (Georgieva et al., 2021). Based on a survey conducted in eleven countries, Georgieva et al. (2021) found that, in Finland, the self-reported level of compliance with measures intended to contain the pandemic was high by international standards. Finnish residents were also quite satisfied with the way their government had handled the pandemic and reported high levels of trust in their political leaders regarding the issue.

A mini-public regarding the restrictions and guidelines related to containing the COVID-19 pandemic was held online during one weekend, March 13th and 14th, 2021. The whole event was organized online using a teleconferencing software (Zoom). During the weeks preceding the event, infection rates increased and new restrictions were introduced during February and March (Finnish Institute for Health and Welfare, 2021b). The primary aim of the mini-public was to examine the impact of expert hearings in a deliberative process, and the management of the COVID-19 situation provided a topical and concrete context for the research. The mini-public was primarily motivated by scientific objectives and was not directly linked to policy processes. However, the research team prepared a non-technical research report on the participants’ views, and a press release based on it was distributed to the media.

### The recruitment of participants

The recruitment to the mini-public started in February 2021 with a post card that was posted to a random sample (*n* = 6000) of Finnish residents aged between 18 and 80. The post card included an invitation to the mini-public, alongside basic information about the event and the monetary reward (75 euros) to which the attendees would be entitled. Respondents were also informed that the mini-public would be organized for research purposes and that the researchers would produce a report concerning the deliberations and participants’ views on the issue, which would be published after the event. No allusion was made to direct impact on or involvement in actual policy processes.

Those willing to participate were asked to fill in a recruitment survey online (*T*1). The online recruitment survey included questions concerning the respondents’ evaluations of the policies that the government had introduced to confine the pandemic, attitudes towards particular restrictions and recommendations, as well as questions about basic socio-economic background variables and political orientation.

Of the sample, 261 (4.4%) filled in the survey, and of these, 163 (62.5% of the respondents) declared that they would volunteer as participants in the mini-public. All volunteers were invited to the mini-public. Of these, 80 confirmed their participation and 74 showed up to deliberate on the first day of the mini-public. Two participants quit during the first day and another two did not show up to deliberate on the second day. Therefore, a total of 70 persons participated for the whole duration of the mini-public.

Despite the relatively low response rate, the mini-public brought together a diverse group of individuals that corresponded well to the national-level demographic composition in terms of gender, age group, and native language. No further selection was made based on responses to attitudinal questions in the recruitment survey because such selection could have made the mini-public excessively small. The detailed description of the Jury’s demographic composition and its comparison to national-level population statistics are presented in Appendix 1. The gender distribution was similar to that of the whole country. Middle-aged people were somewhat overrepresented in the mini-public, whereas those aged 25–34 and the oldest age groups were underrepresented. However, the age distribution of the participants reflects the general age distribution of Finland reasonably well, which is important since older people are more likely to have more severe symptoms of COVID-19, while younger age groups with, on average, a larger number of social contacts are more likely to contract the disease. Moreover, the measures to contain the pandemic have affected the various age groups in different ways.

Highly educated people were strongly overrepresented in the mini-public. This overrepresentation is most likely due, in part, to the technical requirements related to online deliberation. More generally, education has been identified as a consistent predictor of political participation (Persson, 2015), and deliberative mini-publics do not seem to be immune to this distortion. Regarding geographical representativeness, the mini-public had participants from 14 regions out of 19. Notably, the region of Uusimaa, which contains the capital city of Helsinki, was strongly overrepresented. This is could be due to the sociodemographic profile of the region, especially the above-average share of highly educated people, and the fact that the region had experienced the strictest containment measures. Although not all regions were covered, there was notable variation in terms of the COVID-19 situation and the stringency of containment measures in the regions that were represented. Hence, it is reasonable to assume that participants’ personal experiences regarding containment measures varied.

### The experimental procedure

The deliberative process was held over the course of two days, on 13th and 14th of March, and lasted about four hours each day. The participants were tasked with formulating questions for four experts, listening to their responses, and deliberating on the issue in small groups after the expert hearings. Before the deliberative process, participants were divided into two treatment groups (A and B; see below) that deliberated in simultaneous but separate sessions, without having any contact with each other.

Four experts were being heard during the mini-public. Three of the experts were male, and one of them was female. All of them were around the same age and experienced in their profession. Both treatment groups listened to two pairs of experts but in a different order. One pair of experts consisted of a legal expert and a social scientist, the other pair included healthcare professionals. On the first day, treatment group A (*n* = 35) posed questions to the experts about legal and social issues. On the second day, the same treatment group posed questions to the experts in public health. For treatment group B (*n* = 35), the order of the expert hearings was reversed.

Both treatments were divided into five small groups consisting of six to eight participants and a trained facilitator, whose task was to supervise the adherence to the rules of deliberation. For both treatment groups, the first day of the mini-public started with a short introduction to the norms and rules of deliberation, as well as an overview of the schedule of the mini-public. Then, participants watched short video presentations of the two experts to be heard. The short presentations focused on the experts’ professional credentials and their expertise on the COVID-19 pandemic in particular.

Treatment group A was first asked to gather information from two experts: a university professor of public law specializing in the effects of the pandemic on the Finnish legal system, and a research professor in sociology studying the social impacts of the pandemic, e.g. welfare, inequalities, and mental health. At the same time, treatment group B listened to a medical superintendent in charge of responses to the pandemic in a Finnish city, and a research professor and epidemiologist specializing in national health and statistics on the pandemic. In addition to scientific expertise, the experts had practical experience from designing and implementing measures to mitigate the COVID-19 pandemic. More precisely, the public law professor was one of the key experts heard in drafting the pandemic legislation in Finland, and the medical superintendent was involved in implementing the legislation.

After the video presentations, each small group was asked to formulate questions for the two experts just introduced. The participants were instructed to focus on the experts’ field of specialization and formulate questions that these particular experts would likely be able to answer. Each group was expected to formulate four questions and put these questions into an order of priority. The first two questions would be presented to the experts, while the other two would be backup in case of overlapping questions from other groups. Throughout the formulation of the questions, the facilitators encouraged people to work as a group and identify common points of interest, in addition of overseeing that the discussion remained respectful and inclusive. Votes were taken if there were disagreements on the content or order of the questions.

The questions formulated in small groups were posed to the experts in a plenary where all participants of the treatment group were present. The facilitators presented their small groups’ questions, and both experts had around two minutes to answer each question. After the main questions, backup questions were posed. The number of questions asked during the Q&A sessions was between 10 and 13. During the small group discussions after a lunch break, participants deliberated on the issue and the expert information they had received. More specifically, the participants were asked to express their thoughts and views after they had heard the experts’ answers. Finally, the participants returned to the main sessions where they received directions to fill in a survey (*T*2) and continue deliberations the next day.

The second day of the mini-public followed the same schedule as the first day. The treatment groups remained the same, but the experts rotated; treatment group A interacted with health experts, while treatment group B listened to experts in law and social sciences. The mini-public ended with a final survey (*T*3). The *T*2 and *T*3 surveys included questions concerning general evaluations of the governmental response to the pandemic, as well as specific measures to contain it. In addition, there were questions measuring participants’ factual knowledge related to the pandemic, trust in different sources of information, and opinions of the experts’ answers. Together, *T*1, *T*2 and *T*3 allow for quantitative analyses on how participants’ opinions and attitudes developed over the course of the event.

No separate survey was carried out at the beginning of deliberation and recruitment survey *T*1 was used as baseline in order to minimize anchoring effects (Gehlbach & Barge, 2012). All 70 participants who took part during both days also completed the surveys *T*2 and *T*3.

In addition to *T*1, *T*2 and *T*3, a separate control survey was administered around the same time the mini-public took place. This was done in order to get a grasp of the opinion changes that might be caused by the evolving coronavirus situation, regardless of deliberation. A random sample of survey invitations (*n* = 1000) yielded 131 answers between 10 and 19 March, a period during which no major shifts in public debate regarding the virus situation could be identified. Although the small *n* does not allow for population-level statistical deduction, the control survey responses give a rough estimate of the general attitudes prevailing at the time of the mini-public.

The experimental procedure and the surveys are summarized in Table 1.

**Table 1:** The experimental procedure

Experimental group	Treatment A (5 groups, *n* = 35)	Treatment B (5 groups, *n* = 35)	
*Recruitment survey T1 (February 2021)*			
Day 1 (Saturday 13 March)	Legal/social experts	Health experts	*Control survey* (10–19 March 2021, *n* = 131)
*Survey T2*			
Day 2 (Sunday 14 March)	Health experts	Legal/social experts	
*Survey T*3			

Although expert hearings in the mini-public took place in a plenary, the idea was to allow deliberation both on the formulation of questions and on the experts’ responses to those questions. The expert hearings dealt with the questions that the participants formulated in small groups, which allowed participants to discuss experts’ credentials and to pose relevant questions given the areas of expertise. Appendix 2 lists the questions that were posed to experts during the Q&A. In addition, the participants had opportunities to exchange their views on the experts’ responses, which should have encouraged critical reflection on expert information and correction of biases in the interpretation of the evidence.

Based on participants’ overall evaluations in *T*3, the guiding principles of deliberation were well adhered to in the mini-public. 97% agreed completely with the statement “other people’s views were respected and listened to”, 44% agreed completely and 35% partially with the statement “a diversity of opinions were represented in the discussion” and 90% agreed completely with the statement “the moderators of the discussion were impartial”. In contrast, 96% disagreed completely with the statement “during the discussions, I felt pressure to agree with the others on something I wasn’t quite sure about.” When it comes to the statement “some participants dominated the discussion too much,” 97% disagreed completely.

## Results

In order to understand the impact of the field of expertise on participants' opinions, we first explore whether the experts’ fields of specialization played a role in terms of what kinds of questions participants formulated for the experts, and how these questions were answered. Were there questions that were typically directed to one type of expert but not to the other? This is important since the types of questions posed to the experts could potentially influence the ways in which participants framed the governmental policy responses to the pandemic and, consequently, their attitudes towards those policies. Similarly, we should establish how the statements of the experts themselves framed the issue.

An overview of the questions in Appendix 2 reveals that irrespective of treatment, some themes were prevalent solely in the questions for the legal and social experts and some only in the questions for the health experts. Questions related to the Emergency Powers Act, which was temporarily in force in the spring of 2020, law drafting, the (in)flexibility of jurisdiction and the formulation process of COVID-19 restrictions were directed to the legal and social experts, but not to the health experts. Furthermore, most of the questions related to the adverse side-effects of restriction measures were posed to the legal and social experts. In contrast, questions regarding vaccination coverage, COVID-19 variants and contamination routes of the virus were posed only to the health experts. There were some overlapping themes including, for example, vaccination order, measuring the restrictions’ impacts on welfare, and a potential curfew (that the government was preparing) and its justifications. While the field of expertise did not fully determine the themes of any Q&A session, it clearly influenced the questions posed.

A look at the expert answers lends more support to the assumption that field of expertise affected the framing of the issue. The social and legal experts expressed more criticism towards strict, mandatory containment measures and underlined the importance of basic rights, describing how they are safeguarded in the Finnish legal system. They also highlighted how the pandemic and containment measures had exacerbated existing structural inequalities, and how it was hard to strike a balance between all the harmful effects stemming from the disease itself and the measures to curb it. Health experts, on the other hand, expressed more worry about people not following the instructions and regulations, and emphasized that a multiplicity of efficient measures were needed. They approached legislation more in terms of its instrumental value in disease containment. The differences in experts’ emphases is probably partly due to the different nature of the questions asked.

Some common themes emerged as well. In all hearings, experts posited, e.g. that Finland had done relatively well in fighting the virus compared to other countries, and that information provision had been challenging in the quickly evolving situation. Nevertheless, the answers provided participants with two somewhat distinct viewpoints to the issues of disease containment.

In sum, fields of expertise were not insignificant to the participants since at least they seem to have framed the question formulation. In addition, topics that were emphasized in the answers differed depending on the experts’ field. Based on these observations only it is not possible to say whether the questions or the expert answers determined the themes of the small-group discussions. A detailed analysis of the discussions is, however, outside the scope of this study as we are primarily concerned with the kinds of attitudinal changes that followed the discussions.

We now turn to the more direct testing of our hypotheses 1–3 regarding the impact of the fields of expertise on the development of opinions. Participants’ attitudes towards COVID-19 policies were inspected by asking participants whether they agreed or disagreed with certain statements related to the containment of the pandemic. Participants answered the question “What do you think of the following statements?” on a five-point Likert scale (completely agree, partly agree, partly disagree, completely disagree, cannot say). To explore the effects of the expert hearings on participants’ views, we focus on three statements: 1. *Government actions to prevent COVID-19 have unnecessarily violated citizens’ basic rights.* 2. *COVID-19 containment should be based on clear restrictions and orders instead of recommendations.* 3. *The most important thing in fighting the COVID-19 pandemic is to secure the capacity of the healthcare system and prevent new cases, even if that restricts citizens’ daily lives and causes major financial costs.* Three participants did not provide an answer for statement 3 in either *T*2 or *T*3, so these respondents were treated as missing values in the analysis.

Based on H1, we expected that listening to public health experts would make participants more supportive of tight restrictions and thus increase agreement with statements 2 and 3. Further, based on H2, we expected that listening to social/legal experts would make participants more critical towards tight restrictions and thus more supportive of statement 1. H3 regarding discursive path-dependency entails that the experts that the group heard *first* would create a lasting frame for the entire deliberation. Therefore, the attitude changes and differences between treatment groups should be evident, not just in *T*2, but also in *T*3. On the contrary, if the frames created by different kinds of expert information canceled each other out, differences should be visible in *T*2 but not in *T*3.

The observed modest changes in the three items listed in Table 2 indicate partial support for H1 and H2. It seems that the participants’ views on COVID-19 restrictions in Finland did change during the mini-public, but the observed changes are small and lack statistical significance at conventional levels in the case of statements 1 and 3. Regarding statement 2, there was a clear shift towards more support for restrictions instead of recommendations after the first day in treatment group B. This suggests that hearing medical experts did indeed make participants in group B more likely to support tighter restrictions. However, this shift was reversed after hearing the legal and social experts. Therefore, there seems to be no signs that the order of expert hearings played a major part in views measured in *T*3.^1^ Overall, there is very little evidence to support H3, and it seems that the answers heard on the first day did not play a more pronounced role in opinion formation than those heard on the second day.

**Table 2:** The development of participants’ opinions on pandemic policies

	Treatment group							
	A (*N* = 33–35)				B (*N* = 34–35)			
	*T*1	Change * T*1–*T*2	Change * T*2–*T*3	Change * T*1–*T*3	*T*1	Change * T*1–*T*2	Change * T*2–*T*3	Change * T*1–*T*3
*What do you think of the following statements? 1 = Completely disagree, 5 = Completely agree*								
1. Government actions to prevent COVID-19 have unnecessarily violated citizens’ basic rights	1.69 (0.20)	0.20 (0.17)	− 0.09 (0.21)	0.11 (0.19)	2.00 (0.20)	0.06 (0.15)	− 0.00 (0.21)	0.06 (0.19)
2. COVID-19 containment should be based on clear restrictions and orders instead of recommendations	3.91 (0.20)	0.11 (0.16)	0.03 (0.18)	0.14 (0.16)	3.43 (0.23)	0.46* (0.21)	− 0.63** (0.22)	− 0.17 (0.25)
3. The most important thing in fighting the COVID-19 pandemic is to secure the capacity of the healthcare system and prevent new cases, even if that restricts citizens’ daily lives and causes major financial costsª	4.12 (0.16)	0.09 (0.15)	0.09 (0.17)	0.21 (0.16)	3.89 (0.19)	0.17 (0.17)	0.12 (0.13)	0.24^†^ (0.13)

ªThe differences between total change *T*1–*T*3 and sum of changes *T*1–*T*2 + *T*2–*T*3 are caused by missing values

^†^*p* < 0.1, *p < 0.05; **p < 0.01, based on two-sided paired sample t-tests. Standard errors in parentheses

To further test the hypotheses, we inspect participants’ support for some restrictions that had been in place in Finland at some point (most restrictions with varying strictness levels) since the start of the pandemic. In *T*1–* T*3, participants were asked how acceptable they found a particular restriction on a scale from 0 to 10, where 0 meant “not at all acceptable” and 10 meant “fully acceptable”. We focus on the following restrictions: restrictions on movement within the country, limitations for public events, closure of schools, restrictions to opening hours of bars and restaurants, and closure of certain public spaces.

For the five restriction variables, we first performed a factor analysis to establish whether we could meaningfully discuss support for restrictions as a general category. The analysis returned high values for a KMO and Bartlett’s test (*T*1 = 0.858, *p* < 0.001; *T*2 = 0.772, *p* < 0.001; *T*3 = 0.723, *p* < 0.001) and all variables loaded to a single dimension in *T*1, *T*2 as well as *T*3. Attitudes to different restrictions were also highly and significantly correlated.^2^ Based on the results of the factor analysis, we combined the five restriction variables into a single “support-for-restrictions index” ranging from 0 to 50, where a higher score denotes more favorable view towards restrictions. The mean of the index in *T*1 for all participants is 36.24, and the standard deviation is 11.46 (median 39, range 0–50).

The initial level of and changes in the restriction support index are reported in Table 3. As can be seen, the level of support for restrictions is somewhat lower in treatment group B than group A, and this remained the case throughout the weekend. However, in both treatment groups, the changes in support are similar in magnitude and direction: support for restrictions increased from *T*1 to *T*2 and, to a somewhat smaller extent, from *T*2 to *T*3. Part of the explanation for the increased support for tight restrictions from *T*1 to *T*2 is probably the fact that the overall COVID-19 situation worsened between these two measurement points. This assumption is supported by results from the control survey, in which the support for restrictions index yielded a value of 38.91. By contrast, the same value among the recruitment survey respondents who did not take part in the discussion was 36.14.

**Table 3:** The development of support for restrictions

	*T*1	Change * T*1–*T*2	Change * T*2–*T*3	Change * T*1–*T*3
*Support for restrictions index (min. 0, max. 50)*				
All	36.24 (1.37)	2.07* (0.98)	0.50 (0.70)	2.57** (0.80)
Treatment A	37.45 (2.03)	2.40^†^ (1.26)	0.34 (0.98)	2.74** (0.89)
Treatment B	35.03 (1.85)	1.74 (1.51)	0.66 (0.99)	2.40^†^ (1.32)

^†^*p* < 0.1, *p < 0.05; **p < 0.01, based on two-sided paired sample t-tests. Standard errors in parentheses

The index increase from *T*1 to *T*2 was 0.66 points higher for group A than for group B, whereas, from *T*2 to *T*3, the increase for group B is 0.31 points higher. These differences are not statistically significant, however. The results indicate that the field of expertise had no systematic impact on overall support of restrictions and thus yields no support for H1 and H2 and, consequently, H3 does not gain support either.

Based on our analysis, none of the three hypotheses presented in this study receive consistent support. This suggests that neither the fields of experts heard nor the order in which they were heard were influential in opinion change among the participants of the mini-public.

### Participants’ evaluations of expert answers

In the last part of analysis, we look at how participants evaluated experts’ answers at the end of both days of deliberation. This evaluation was done in order to assess whether the participants perceived systematic differences between experts in terms of the quality of their answers. For example, a situation where participants in both treatments would rate the health experts’ answers more favorably than the legal and social experts’ answers would be relevant when measuring opinion change and could help determine why opinions developed the way they did.

The results in Table 4 show that there were no systematic differences between the two groups’ evaluations of experts’ answers. All participants ranked the experts’ answers more positively on the second day on all measures: usefulness, diversity, justification, depth, assertiveness and relevance. This was true for both treatment groups, but even more pronounced in treatment group B. However, as both treatment groups rated the experts’ answers higher on the second day, regardless of the type of experts they were, there is no clear evidence of either pair of experts performing better than the other.

**Table 4:** Participants’ evaluations of experts’ answers

Treatment	All (*N* = 68–70)			A (*N* = 34–35)			B (*N* = 34–35)		
	*T*2	*T*3	Change	*T*2	*T*3	Change	*T*2	*T*3	Change
*How would you rate today’s expert answers? To what extent were they…? 1 = Not at all; 5 = Very much*									
Useful	3.54	3.87	0.33**	3.60	3.74	0.14	3.49	4.00	0.51**
Diverse	3.46	3.91	0.46***	3.43	3.66	0.23	3.49	4.17	0.69***
Well-founded	3.86	4.13	0.27**	3.83	3.94	0.12	3.89	4.31	0.43**
Profound	3.00	3.57	0.57***	3.03	3.32	0.29*	2.97	3.80	0.83***
Convincing	3.52	3.94	0.42***	3.41	3.76	0.35**	3.63	4.11	0.49**
Relevant	3.49	4.04	0.56***	3.41	4.03	0.62***	3.56	4.06	0.50**

*p < 0.05; **p < 0.01;***p < 0.001, based on two-sided paired sample T-test

This finding suggests that the communication between the experts and participants improved over the course of the event. This can be due to participants becoming more experienced in crafting questions and listening to experts’ responses, but also because the experts became more accustomed to answering the kind of questions they were presented with. In the mini-public, the experts did not read the questions beforehand or prepare their answers; rather, they answered them right away.

On the other hand, the results might also reflect the overall satisfaction among the participants at the time the deliberations ended. It is possible that after the first day, when the participants had only listened to one pair of experts, they developed sentiments that they still needed to know more about the matter, and after the second day, they had the chance to acquire more information and fill those self-identified gaps in their knowledge. Therefore, after getting to hear different sides of the matter, they gave more positive evaluations. The measurements of participants’ factual knowledge before and after the event give some support to this assumption. Whereas the initial level of knowledge in group A was high to begin with and did not change during the event, treatment group B experienced a significant increase in knowledge (see Appendix 3). Additionally, in both treatment groups the number of participants who reported that they understood the subject somewhat or much better than before increased from *T*2 (54.3% in both groups) to *T*3 (71.4% in group A and 85.7% in group B).^3^

## Conclusion

During the COVID-19 pandemic, expert opinions about desirable responses have varied considerably. This is not only because in the beginning little was known about the virus itself, but also because different experts have viewed the same problems from different angles. Meanwhile, experts have had an important role in planning and shaping national policy responses to the pandemic. In general, the way in which most democracies have dealt with the outbreak of the COVID-19 pandemic and how they have tried to mitigate impacts of the crisis can be seen as examples of how technocratic tendencies are developing in representative systems.

The aim of this study was to explore whether the outcomes of mini-publics may be affected by the specific field of the experts heard, and whether the possible frames created by expert information are durable. Mini-publics have been regarded as institutions that can help bridge the knowledge gap between lay persons and experts in decision making, and democratize expertise (see, for example, Brown, 2014; Moore, 2017; Smith, 2009). This expectation is not fulfilled if participants become too deferential to scientific authority, only receive one-sided viewpoints from experts, or do not critically weigh the information they have received.

The results seem to dispel some of the concerns regarding the role of expert information and the risks of expert domination in deliberative mini-publics. Opinions did change over the course of the event; that is, both treatment groups became slightly more supportive of restrictions introduced. However, there seems to be no indication that either the academic field of experts heard or the order of expert hearings had any significant impact on opinion change. In other words, while the fields of experts seem to have framed the deliberative process to some extent, these frames did not seem to guide the deliberations in ways that would result in systematic changes in opinion.

There is at least one design feature in this mini-public which may explain why we found no such systematic impact. Namely, the participants were tasked with crafting questions to experts and were therefore able to receive answers to precisely the questions that they had in mind. No briefing materials or plenary lectures, which are common in deliberative mini-publics, were utilized (see Roberts et al., 2020). It is moreover likely that in the Q&A sessions, especially those held online, the personal charisma and presentation skills of experts do not have the same influence on perceived assertiveness of expert information as would be in the case with carefully prepared and rehearsed plenaries.

Our results are in line with the view that mini-publics provide good opportunities for public scrutiny of expert information. Deliberative mini-publics can be designed to emulate the ideal role for expertise in public deliberation, which Moore (2017, 34) describes as “informing democratic opinion, but not manipulating it; empowering democratic will, but not dominating it”. In evidence gathering through Q&A sessions, the participants themselves had an active role in determining what they wanted to know, instead of the organizers or experts determining what they needed to know. In addition, the chosen procedure of expert hearing might have cultivated critical thinking in a way that another procedure, for example, a plenary lecture or an information sheet, might not have. Nonetheless, we acknowledge the need for future research on the impact of expert information in mini-publics. As indicated above, future studies could profitably address the effects that alternative formats of expert hearings potentially have. Moreover, the theme of our mini-public was the management of a crisis that was still quite acute when the discussions took place. Future studies could also address more long-lasting issues with respect to which opinions have had more time to settle.

In sum, our findings do seem to suggest that interactive modes of expert hearings in mini-publics are not particularly prone to expert domination, and in this respect, they may have merits in comparison with other types of expert hearing processes.

## Appendix 1: Demographic composition of the mini-public

**Table:** 

	Finland 2019^a^	Participants	
	%	*n*	%
*Gender*			
Female	51	36	51
Male, other and no information	49	34	49
*Age group*			
18–24	10	8	11
25–34	16	8	11
35–44	16	11	16
45–54	15	15	21
55–64	16	14	20
65–74	16	11	16
75+	12	3	4
*Native language*			
Finnish	88	63	90
Swedish	5	3	4
Other	7	4	6
*Region*			
Uusimaa	31	35	50
Other regions^b^	69	35	50
*Education level*			
Low level	23	4	6
Intermediate level	54	20	29
High level	23	45	64
Other/no information	0	1	1
Total	100	70	100

^a^Statistics Finland (2021).

^b^Participants from the following regions were present in the mini-public: South Karelia, South Ostrobothnia, South Savo, Kanta-Häme, Central Finland, Kymenlaakso, Pirkanmaa, North Karelia, North Ostrobothnia, North Savo, Päijät-Häme, Satakunta, Uusimaa and Southwest Finland. The regions of Åland, Kainuu, Central Ostrobothnia, Lapland and Ostrobothnia were not represented in the assembly.

## Appendix 2: Questions posed to experts by participants

**Table:** 

Treatment A (legal and social experts)	Treatment B (health experts)
*Saturday*	
In what way and how strongly can fundamental and individual rights be limited in Finland? How do Finnish fundamental rights and laws differ from other Western states? Is Finnish legislation so much stricter than in other Western countries that it makes it more difficult to impose the restrictions here? Or is the legislation just interpreted much more strictly here than in other Western countries?	How much has the pandemic impacted the total mortality in Finland? At the beginning of the pandemic this didn’t seem to have changed, but has the rate of total mortality now changed as the coronavirus situation has progressed? How many people have been reinfected in total or proportionally in Finland? How many Finns have developed immunity against this disease? Is there also a difference in age groups, because sometimes the message seems to have been that corona only affects the elderly, but young people have also gotten ill.
When can the pandemic be declared to be over? What are the criteria to be met for the pandemic no longer to exist? This is especially important for entrepreneurs because what will happen if corona continues for another 20 years? How should we prepare for this?	How much can the attitudes and motives of a population be influenced to make people comply more with instructions and restrictions? Is it also possible to see some working examples from other countries here, which could also be adopted in Finland, and so cooperate internationally in the matter?
Why wasn’t drafting of legislation implemented during the ‘calmer phase’ in 2020, when the accelerating of a disease situation was already known?	Where does the idea of a curfew come from? Does it have a medical and statistical background? And what would a curfew mean?
Has the idea of the negative effects of corona restrictions on well-being impacted their formulation, and has how the restrictions affect well-being been studied?	Where does the disease in reality occur, i.e. where are the routes of transmission and from where does the disease spread home? What do we know about virus variants and their spread?
New Zealand’s and Australia’s models: What separates Finnish regulations from the legislation of these countries and does the EU cause problems for closing borders? Is the Finnish legislation rigid; why, for example, have no borders been closed?	The potential of artificial intelligence in disease management: where is this data obtained from, in what form should it be and how can it be shared and analyzed? Artificial intelligence first detected a deviation of COVID-19 in China and data collection has been effective in China. How can data collection be improved?
How could young people be supported more, so that they would be able to continue their studies? Has research been conducted, and is research being conducted on whether socioeconomic status affects people’s coping in the times of corona?	The Finnish vaccine seems good, effective and simple. Why is it not being deployed more quickly into use? How is the vaccine funded, and how will it be manufactured?
How has the responsibility of imposing COVID-19 restrictions changed since last spring, and what impacts will it have on the extent of COVID-19 restrictions?	How do you, chief physician [one of the experts], see the future of the viruses: can we predict whether pandemics will be more common in future? When does COVID-19 become like seasonal influenza, and we can return to so-called normal life?
Taking vulnerable people into account in COVID-19 restrictions, are there any other ways to lift the restrictions than the vaccine?	How high should vaccine coverage be in order for it to contain the spread of the virus? When is herd immunity achieved if it is aimed at, or do we only seek to protect high-risk groups through vaccination?
Can the Constitution and its relation to the Emergency Powers Act be reconsidered in that it would be more flexible and would permit, for example, the closure of borders in crisis situations such as this?	Mobility and mandatory face masks: do statistics support a curfew more than a requirement to wear a face mask? Is there evidence of this from other countries where a curfew is in place? Does it matter if the restrictions on mobility are in place all day or, for example, only in the evening?
Could the COVID-19 vaccination programme be refined, for example, by first vaccinating in areas where the spread of the virus is rapid?	Has the Koronavilkku contact tracing application been useful, for example, in tracing infections or something else? Does THL [Finnish Institute for Health and Welfare] follow the user rate of the application?
	The new virus variants appear to spread faster. Is droplet infection still the most important form of transmission? Is it passed on from surfaces and the air, outdoors and indoors, how easily? And what is the benefit of using different masks in the light of the spread of new virus variants?
	What indicators are being developed to examine, currently or 5–10 years from now, the health and well-being of young people and the progress of their studies?

**Table:** 

Treatment A (health experts)	Treatment B (legal and social experts)
*Sunday*	
What is the planned vaccination order or prioritization, and who decides upon it? In the beginning, there were age-based and front-line employees, and will there be changes to the original plan?	How has the time with COVID-19 affected those working in the social welfare and healthcare sectors when the work atmosphere has been oppressive and nervous? Will there be enough employees in the future when 90% of nurses would like to change their field of work?
All working-aged people willing to take the vaccine would get the vaccination by July. Is this realistic at the moment?	Is it still possible to tighten restrictions so that companies don’t go bankrupt? Why are the various forms of aid to companies not tied to different restrictive measures so that the aid follows the restrictions immediately? This is also linked to border control
The legislation already allows for, for example, mandatory testing at borders, but why haven’t it been implemented yet? Is it political decision makers or officials (Finnish Institute for Health and Welfare or the regional state administrative agency) who make these decisions?	Are young people’s mental well-being and social skills taken into account in COVID-19 restrictions? Have experts had their voices heard on these issues, or are mortality rates and the economy prioritized within the scope of issues that are focused on?
What are the arguments for the curfew, and do you think it would be useful, especially when the country is so sparsely populated? Should other measures, such as stricter testing at borders, be applied before it is introduced?	Increasing inequality. How much has it increased and what should be done to prevent it? … related to the COVID-19 period
Have your statistical reviews taken into account the potential resistance of virus variants to vaccines and can they be predicted?	From a jurisdictional point of view, who decides when restrictive measures, such as the Emergency Powers Act or closures, such as the Uusimaa region closure, are introduced?
Where would it be sensible to test asymptomatic people within the borders of Finland, and to which groups would the testing of asymptomatic people be targeted?	The economic cost of an epidemic can be calculated retrospectively, but what indicators exist to measure the impact on social and mental well-being? If indicators and data already exist, are these used and in what way?
Now that 10% of citizens have been vaccinated, they have to wait a long time before everybody else is vaccinated, too. Can restrictions be lifted gradually, or will all restrictions be maintained until everyone is vaccinated? Can we start lifting restrictions once high-risk groups are vaccinated?	Has the impact of hobbies on well-being and health been studied, and what importance do hobbies and not being able to pursue one’s hobbies have on, for example, the consequences [of the pandemic], also in the case of adults?
How are decisions made when the order of vaccination is considered? What are the factors behind these decisions? How could people who are at risk of getting infected at work get the vaccine earlier?	On what basis would a curfew be enforced, and how would the areas for curfew be selected? Doesn’t it put people in an unequal position, for example, in terms of household size (for example, people living alone should only meet one person)?
During the epidemic, a lot of endurance has been required from healthcare workers, and many have been transferred to new tasks. Has this jeopardized healthcare? How big of a ‘healthcare deficit’ is there?	Would it be possible to make legislation more flexible by creating so-called pandemic legislation for the future?
What role do you think the Finnish Institution for Health and Welfare (THL) should play in informing the public; would a ‘corona fist’ [a concerted group of officials and politicians for administering measures related to the virus] have made communication easier?	How has the change in the way the corona situation has been presented in the media, in the early days of the pandemic compared to today’s situation, affected general coping/mental health (from cheers to blame)?
Has the impact of hobbies on well-being and health been studied, and how much weight do hobbies and not being able to pursue one’s hobbies have in the aftermath [of the virus]?	What are the risks associated with the introduction of the Emergency Powers Act?
How do you view how the corona-related communication has worked, both professionally and personally?	
Have the closures worked; would it be possible to apply them more at a local level?	

## Appendix 3: Participants’ level of knowledge

Level of knowledge was measured in *T*1 and *T*3 using different statements that concerned COVID-19 containment measures and the disease itself. Five of the statements were true and five were false. Participants were asked to evaluate independently whether a statement was definitely true, probably true, probably false or definitely false or whether they did not know. For the analysis, the answers were scored and recoded from 0 to 4 so that a higher score corresponded with the correctness and level of certainty of an answer, e.g. if a statement was true and a respondent evaluated it as “definitely true”, they would receive four points, two points if they answered “don’t know” and zero points if they answered “definitely false”. The scores from all statements were then combined to form a level of knowledge index, with a maximum value of 40. The knowledge index changes are reported below.

**Table:** 

Treatment group	*T*1	*T*3	Change *T*1–*T3*
*Level of knowledge index, mean*			
All (*N* = 69)	32.10 (0.53)	32.77 (0.55)	0.70^†^ (0.41)
A (*N* = 34)	32.91 (0.73)	33.03 (0.59)	0.12 (0.61)
B (*N* = 35)	31.26 (0.75)	32.51 (0.93)	1.26* (0.54)

^†^*p* < 0.1, *p < 0.05, based on two-sided paired-samples T-test. Standard errors in parentheses.

The average knowledge index in *T*1 among respondents who did not take part in the deliberation was 30.99. The level of knowledge observed in the control group was 30.77.

## Footnotes

1 Nonparametric tests (paired samples Wilcoxon tests) produced results that were substantively the same.

2 Extraction criterion: Eigenvalue = 1; component loadings: *T*1 = 0.766–0.857; *T*2 = 0.660–0.873; *T*3 = 0.726–0.806. Correlations: *T*1 = 0.512–0.738; *T*2 = 0.353–0.733; *T*3 = 0.370–0.710; *p* < 0.001.

3 How much more do you know about restrictions and recommendations for coronavirus containment after today’s deliberation? 1 = The subject confuses me more than before; 2 = I understand it as well as before; 3 = I understand it somewhat better than before; 4 = I understand the subject much better than before.
